# Post - effects of obstetric fistula in Uganda; a case study of fistula survivors in KITOVU mission hospital (MASAKA), Uganda

**DOI:** 10.1186/s12889-019-7023-7

**Published:** 2019-06-06

**Authors:** John Bosco Bomboka, Mary Gorrethy N-Mboowa, Jennifer Nakilembe

**Affiliations:** 1 Clarke International University (Former International Health Sciences University), Kampala, Uganda; 2 Clarke International University (Former International Health Sciences University), Uganda Virus Research Institute MUII-PLUS, Kampala, Uganda; 3 Department of Population Studies, School of Statistics and Planning, Makerere University, Kampala, Uganda

**Keywords:** Fistula, Obstetric, Post effects of fistula, Survivors, Experiences, Uganda

## Abstract

**Background:**

Obstetrical fistula (OF) is a public health challenge that is among the previously neglected components of maternal health in the developing world. The condition, which in the recent past has increasingly drawn more attention from the public, has a devastating impact on the health and wellbeing of both women and girls worldwide. The most common cause of obstetric fistula in developing countries is prolonged obstructed labor affecting approximately 2 million women and girls across Africa and Asia.

The objective of this study was to examine the post-effects of fistula and reintegration strategies of fistula survivors in Uganda.

**Methods:**

A descriptive case study design was used to collect data from women aged 15–49 years who had experienced OF and been successfully treated/repaired. Data collection was aided by in-depth interview guides designed for collecting qualitative data which was analyzed using thematic and content analysis.

**Results:**

The study results showed that 45.6% were aged 18–24 years, 43% had only primary level education and 55.7% of the women were married. Fistula survivors continue to suffer from shame, rejection, isolation and stigma, trauma and disgrace among other effects even after successful repair/surgery. Some of the reintegration strategies for fistula survivors include; seeking for successful repair, remarriage and relocation from their parent communities to new environments.

**Conclusion:**

In addition to capacity building, changing attitudes and strengthening the health system, a comprehensive and holistic fistula care approach is required to facilitate the reintegration process and restoration of women dignity.

**Electronic supplementary material:**

The online version of this article (10.1186/s12889-019-7023-7) contains supplementary material, which is available to authorized users.

## Background

Obstetric fistula (OF) is a complication of childbirth occurring almost exclusively in developing countries. This abnormality results from prolonged obstructed labour which is usually associated with delays in seeking or receiving appropriate emergency obstetric care especially among young adolescent mothers [1, 2]. Other causes of obstetric fistula include among others; destructive deliveries, caesarean section with or without hysterectomy and symphysiotomy [3].

This condition is increasingly drawing more attention among the previously neglected components of maternal health in the developing world because of its devastating impact on the health and wellbeing of those living with it [4]. It is one of the most severe childbirth injuries which leaves an opening between either the “bladder and the vagina” (vesico-vaginal fistula) or the “rectum and the vagina” (recto- vaginal fistula) resulting into urine or faecal incontinence respectively [5]. It is estimated that approximately 2 million women and girls across Africa and Asia are living with untreated obstetric fistula and up to 100,000 new cases occur each year worldwide [2]. In Malawi, the prevalence of obstetric fistula is estimated at 1.6 in every 1000 women [6].

In Uganda, the Ministry of Health (MoH) recognized obstetric fistula as a silent morbidity among Ugandan women in 2001 however, strategic measures were not taken until the shocking reports in 2005, where Uganda reported the third-highest prevalence of fistula in the world [7]. It is estimated that in Uganda, 140,000 women were living with fistula by 2009 however; the Uganda Demographic and Health Survey (UDHS) reported a reduction in the prevalence among Ugandan women who had experienced fistula from 3% in 2006 to 2% in 2011. Of these, about 62% of the women suffering from obstetric fistula sought for treatment from the available health facilities [8, 9].

Obstetric fistula is such a devastating condition because it physically and socially disables women [10, 11]. Besides instigating urine and faecal incontinence, obstetric fistula also affects the health, social, economic and psychological well-being of women. Economically, this life-long disability does not only affect the productivity of the woman alone but also that of her household and the community [11–14].

Despite the effects of obstetric fistula on women, the condition can be corrected through surgery and the success rate for the repair has been reported to be more than 80% in different reports [14, 15]. Although surgery can be used to address the physical damage and can have a positive outcomes on women, their families, and communities; little is known about the long-term consequences of the repair on the social, psychosocial, economic and reproductive wellbeing of the survivors in Uganda and the strategies used to successfully re-integrate them back into their families and society.

Identifying the outcomes of fistula repair among survivors is a very important step in assessing the long-term quality of life of women after fistula repair. The study results will aid interventions to address; the non-physical consequences of obstetric fistula among survivors and the strategies for reintegrating them back into their families and society.

## Methods

A descriptive case study design was used to gain insight into the Post-effects/ experiences of fistula survivors. The study which was cross-sectional in nature was conducted among women aged 15–49 years who had experienced OF and successfully received corrective surgery from Kitovu Mission hospital which is located in Masaka district. This cohort was undergoing follow up care from the hospital by the time the data was collected in 2010.

Kitovu hospital is one of the two private, faith-based hospitals under the partnership, Fistula Care Plus. It has a specialised fistula repair clinic and it serves as a referral site for complex fistula repairs. The hospital is also used as a regular camping site for visiting master surgeons where many women with different fistula cases can be repaired at once.

This facility receives patients from different geographical locations with different health related cases/complaints but in this study, only 149 participants were enrolled after meeting the following screening criteria;

- The reported fistula condition/case should have been pregnancy (obstetric) related
- The participant should have received medical and fistula repair/treatment at Kitovu Mission Hospital before March, 2010.
- The repair should have been successful irrespective of the number of attempts (surgeries) made to correct the condition.

Both qualitative and quantitative data collection methods were used.

In order to reach these study participants, the researchers made appointments with the patients using the contact list that was provided by the hospital, and each respondent was interviewed from their home or district of residence.

Data collection was carried out by the principle researcher through face-to-face Key Informant Interviews (KII) aided by a prior designed interview guide [see Additional file 1]. However, phone interviews were carried out for those outside Uganda and others who were within but remotely located in their respective districts (hard to reach areas). The tool was designed with questions that sought to collect information on; the experience and feeling of the respondent, as well as the moral and social support from their family, friends and community before and after surgery. These interviews were conducted either in English or Luganda which are the commonly used languages in Kitovu Mission hospital. Interviews were audiotaped, in order to reduce on data loss, more so in situations where the respondent had little time apportioned for the interview. Transcriptions into English were made thereafter and typed verbatim into Microsoft Word by the interviewer for analysis.

Quantitative data was analyzed using MS Excel and qualitative data was analyzed using thematic and content analysis and some finding are also presented using verbatim quotation.

## Results

A total of 149 women from different geographical locations were enrolled in the study having met all the screening requirements.

Results in Table 1 show that In Uganda, 71.1%, 23.5% and 3.4% of the women came from the central, western and eastern regions respectively. The study participants were coming from more than 20 districts, namely; Masaka, Ssembabule, Lwengo, Bushenyi, Wakiso, Kampala, Mpigi, Isingiro, Mityana, Kalangala, Kiboga, Mbale, Rukingiri, Kyenjojo, Kibaale, Masindi, Buikwe, Bukomansimbi, Kiruhura, Moroto, Kapchorwa, Mukono and Kamwenge among others.

**Table 1:** General Characteristics of studied Population

Characteristic		Frequency (*N* = 149)	Percentage
Residence	Central	106	71.1
	Western	35	23.5
	Eastern	5	3.4
	Rwanda	2	1.3
	Tanzania	1	0.7
Marital status before repair	Single	41	27.5
	Married	83	55.7
	Separated/widowed	25	16.8
Education level	No education	57	38.3
	Primary	64	43.0
	Above primary	28	18.8
Age at Pregnancy (Years)	Less than 18	38	25.5
	18–24	68	45.6
	25–31	33	22.2
	More than 31	10	6.7
Parity at delivery that caused fistula	1	78	52.4
	2	25	16.8
	3–4	24	16.1
	More than 4	22	14.8

The study results further revealed that 45.6% and 27.5% of the respondents were aged 18–24 years and below 18 years respectively. It was also disclosed that 43% of the women had attained primary level education and 55.7% who reported having suffered from OF were married. Additionally, about half (52.4%) of the fistula cases reported occurred during the first pregnancy.

Furthermore, the study revealed that for every ten women (fistula survivors) interviewed, nine had successful operations at first surgery operation and one reported to have undergone more than one operation before her condition was successfully managed. More findings of the study are presented in the sections below.

### Post - effects of obstetric fistula among survivors

This study revealed that a number of women continue to experience stigma, rejection and live in fear arising from the past traumatic experiences of how they were treated by both their family members and the general community the time they suffered from fistula. For those who are rejected and abandoned by close family members including their spouses, they continue living in isolation and consequentially develop an attitude which looks at relationships as meaningless.

*“My attitudes towards men changed and I no longer have the true love for men like the one I had before suffering from fistula. This attitude arose from my husband who ran away from me when I needed him the most when I had fistula…” (Female respondent 26 years old, Masaka)*

*After the operation, my social life improved than when I had fistula, although I still experience stigma to a smaller extent and I even decided to relocate to another place for my residence in order to avoid the gossips and where no one knows about my history (Female respondent 43 years, Mbarara)*

**Fig. 1:**
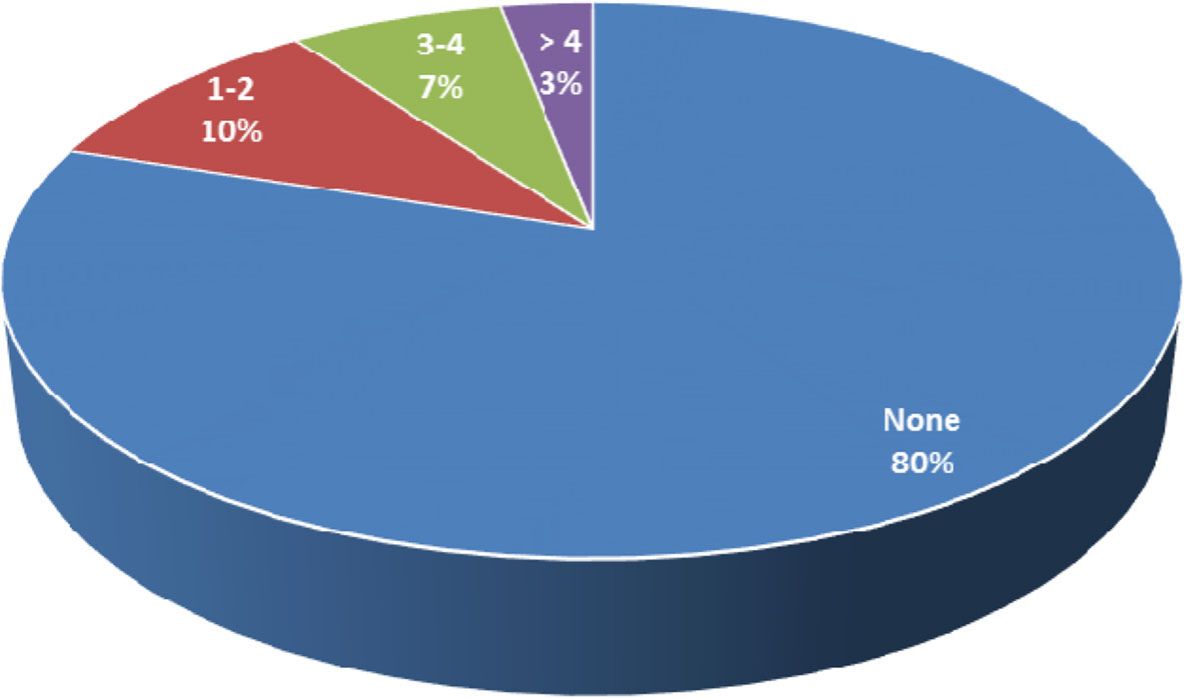
Number of children born to Fistula survivors after repair (*N* = 149)

Many of the fistula survivors still suffer from health-related complications and side effects. Such conditions include; inability to keep urine for a long time, persistent abdominal pains and reproductive consequences such as secondary infertility among others. By secondary infertility we refer to the prolonged delay and or inability of the fistula survivor to conceive and give birth to a child. Results from Fig. 1 show that more than three quarters of the obstetric fistula survivors could not give birth again to another child after the fistula repair. Some of these conditions emanated from the duration the survivor stayed with the fistula as narrated below.

*“… ever since I was operated, I have dated different men and have tried to conceive but in vain…I could have got secondary infertility…” (Female respondent 26 year, Masaka)*

*“My personal health is okay, although my bladder was damaged by fistula due to the so many operations I underwent…I can’t keep urine for a long time… otherwise I might experience severe lower abdominal pain and end up getting infections…” (Female respondent 43 years, Mbarara)*

*“…I can’t have any babies in my life, I got secondary infertility which bothers me so much…” (Female respondent 43 years, Mbarara)*

*“My social life improved greatly than when I had fistula although, I… have spent 6 years with my husband but I can’t give him a child…and I experience a lot of pain when I keep urine for some time” (Female respondent 39 years, Mpigi)*

In as much as some fistula survivors are suffering from secondary infertility, others were able to conceive after successful surgery and have given birth to healthy children despite the fact that majority can only do so by caesarian as reflected in Fig. 1 and the narratives below.

*“After the repair both my social and health life went back to normal and I have no complaints at all…and I thank God that I gave birth to a healthy baby” (Female respondent 39 years, Mpigi)*

*“Since the operation, my personal health is fantastic, no more infections that I used to get when I had fistula…I managed conceive but I can no longer give birth normally but by caesarian” (Female respondent 39 years, Kampala)*

### Re-integration strategies of obstetric fistula survivors

Women suffering from fistula, once given an opportunity to receive corrective repair, eagerly await for the outcome to determine their next step in life. The joy, hope and happiness regained from the successful operation is usually the first step of re-integrating the survivors back into the community as well family members. This is as a result of the transformation they receive individually as far as self-esteem is concerned as narrated by one of the survivors…

*“…the successful repair has brought back the hope that I had lost during the suffering …and I can now stand in the gap and encourage and give hope to those women who are going through the same experience I did go through…” (Female respondent 26 years, Masaka)*

Additionally, community and family’s attitude composed of support, encouragement, joy and positive reception together with celebration and welcome parties before and after operation was used as a coping strategy to help these women get re-integrated back into their families and communities. While some women were not completely in agreement with it, for others it was a pathway to strengthening their relationships.

*My family’s reaction was so welcoming. My boyfriend made a party for me after the successful repair, my mum and my sisters all celebrated for me. I am back to normality and I now work tirelessly with joy (Female respondent 24 years, Wakiso)*

*My family’s reaction when I returned after the repair was positive. My husband (former) was so excited and my mother too was filled with joy. Although my in-laws still despised and stigmatized me, they blamed me of bringing a curse to their family (Female respondent 39 years, Kampala)*

*Off course my family rejoiced when I returned after the repair, if they were so supportive when I had it, what about when I was free from it? My husband threw a party for me which increased my love towards him ((Female respondent 26 years, Mityana)*

From the findings of this study, it appears that remarriage after corrective fistula operations is one of the unlikely scenario especially for those who separate with their partners. However, the findings show that it is one of the means through which the survivors re-integrate back into the community. One of the survivors was rejected by her family and neighbours to the point that even after successfully receiving corrective treatment, she decided not to go back to her family and community for fear of further exclusion and stigmatization. Because of the need for someone to stay besides her to bridge the gap for the loss of the loved ones including the husband, she decided to get married again as coping strategy. She further explained that her current husband is supportive and nice to her. With him, she has managed to comfortably start a new life, eventually reintegrating back into society.

*“… fortunately, after three years of repair, I got remarried to another man and got a baby girl with him something I longed for however, I can’t mention to him about the fistula that I suffered from” (Female respondent 39 years, Kampala)*

For others who encountered a lot of stigma in addition to the trauma from both family and community, the best way they could reintegrate back into society was by relocating from their original communities to new ones thus abandoning their families so as to start a new life where no one around them knows about their past as narrated by one of the respondents.

*“My social life has normalized although it wasn’t easy to adjust to the normal life after the fistula repair. I spent two years after repair without having sex with a man, I was still traumatized. The pain I experienced when I had fistula affected me greatly. I had to abandon my husband and the children that I had given birth to and relocated to Kampala city where no one knew about my history. This was because of the stigma I experienced from almost the entire village and my in-laws during the time I suffered from fistula …” (Female respondent 39 years, Kampala)*

## Discussion

The post-fistula period was a time when survivors unanimously experienced dramatic improvement in their lives. These were manifested through the joy, hope, peace of mind and regained courage to continue living and reintegrating into their own families and communities. After having successful repairs, many women resumed their normal routines and regained their freedom and rights to be, feel a part of, and participate in different activities as it is for other normal women in the society. Previously, these women were denied such simple freedoms and rights enjoyed by other human beings. The hope and esteem regained from the treatment has given others the courage to stand as ambassadors for advocacy in campaigns against fistula.

Nevertheless, many fistula survivors continue to struggle with challenges such as; stigma, relapse, trauma and rejection arising from broken relationships and marriages even after undergoing successful repair(s). This happens more in communities where the perpetrators for such mischief dwell. The challenges mentioned above hamper the ability and capacity of the survivors to participate in community events since it reminds them of the exclusion and pain they went through before. According to Tilahun et al., residual distress and anxiety from misery and social exclusion was reported as a common experience among women whose fistulas had been completely treated [16].

Other women lose the ability to participate in activities which require a lot of strength in fear of re-occurrence of the fistula and or experiencing a lot of pain especially in the lower abdomen due to the weakened muscles. Due to the same reasons, others are unable to hold urine for a long time thus ensuring that they pass it out as soon as need arises. For some, they lose the interest in sex because of the pain and the fragility of their reproductive section and yet for others, having babies becomes a fairy tale because of their inability to conceive arising from secondary infertility. He further gave an account of two women who went as far as avoiding cultural activities that involved intense physical movement, such as funeral rites where women jump and throw themselves on the ground, as well as riding in cars for fear that “the fistula might be untied from the car’s movement”. Several other women conveyed that they “don’t feel free during sex” and were afraid that their husbands would not send them to the hospital for their next delivery. According to Roush, many women are divorced by their husbands and partners, disowned by family, ridiculed by friends and even isolated by health workers. Furthermore, Roush articulated the divorce rate among women who suffer from obstetric fistula as 50 to 89% [17].

Looking at the ways in which the survivors reintegrated into families and community at large, some survivors; relocated to other communities, got married and re-married especially for those who experienced marriage breakdowns and still had a desire for companionship. Some of these women gave birth to more children where possible, and others went back to their families. These reintegration options are majorly attributed to factors such as; family and individual knowledge about fistula and fistula related services, and family and societal attitudes towards the survivor. The study revealed that survivors whose families and community were supportive in one way or the other (be it financially or socially and relationally) did not get many challenges with reintegrating back to their families/homes as compared to their counterparts who experienced rejection, isolation and insults among others. Besides attitude, knowledge about fistula was very key in determining the possibility of the survivor to receiving timely treatment after fistula onset. It is also perceived that for complete healing, the survivors also needed mental therapy and psychosocial rehabilitation on top of physical treatment which focuses on repairing the perforation.

The above results are consistent with the findings by Tiruwork which revealed remarriage and or exit from such a social arrangement as the two major livelihood coping strategies that fistula survivors are using in areas or communities where they have experienced issues to do with rejection and stigma among others [18]. He further suggests that effective fistula treatment consists of a triangulation of services that include; surgery to address physical health, psychosocial therapy to address mental and emotional health and income generating skill building to address economic survival. The comprehensiveness of these treatment(s)/service(s) is intended to facilitate reintegration of the survivor as a productive member of society. Another study conducted by Bangser and colleagues among fistula survivors who had received treatment about 5 years before the actual study in Tanzanian had similar findings. These women felt like their ability to return to work, principally in agriculture and having family support were critical to their reintegration process [19].

The desire of survivors to get married or and remarry and have children is complimented by a study that was carried out by Tilahun, whose results disclosed the desire of women to get married and have children after recovery more so among young women and because of the significant cultural value placed on a woman’s fertility and the boy child [16]. Mselle and his colleagues made a conclusion in their study on fistula repair that restoration of the reproductive capacity of women is one of the essential components to successful reintegration of many women into their communities after surgery in Tanzania [20]. Studies carried out among survivors 1–2 years after OF repair by Drew and colleagues revealed that 45% of these women desired to have additional children in as much as a lower pregnancy prevalence of 10–20% among OF survivors was recorded [21].

In as much as reintegration is a very key component in complete restoration of the survivor, there is a great need for the process to be done in a holistic manner i.e. ascertaining the degree of the preceding factors involved in order to determine whether reintegration is possible or impossible [18]. For example, high levels of stigma accompanied by isolation and alienation from the community, including family members denies survivors an opportunity of playing a social role in their families and communities. This creates a scar of psychological trauma which discourages and frustrates their efforts to smoothly reintegrate back into their families, neighbourhoods and communities. Therefore, because family is the core social institution that critically intersects with the possibility of survivors’ success following postoperative reintegration therapy, there is need to promote social acceptance by close relatives if reintegration is to be made easier.

## Conclusion

Despite the fact that surgical repair has positive outcomes especially towards the physical well-being of the fistula survivor, it doesn’t completely address in totality all the fistula consequences i.e. psychosocial, economic and health for complete recovery. Even after successful surgical repair, women continue to suffer from; shame, rejection, depression, isolation and stigma, trauma and disgrace from their families and community among other social challenges which have continued to affect their quality of life.

Some of the coping strategies that survivors used to reintegrate back into their families and society include; Seeking for information regarding fistula treatment, remarriage (either reuniting with their previous partner or finding a new partner) and relocating to a new community where no one has a record of their past. The reintegration process was highly dependent on; family and community attitude towards the survivor and their knowledge about fistula.

We recommend that using a comprehensive and holistic fistula care approach will help to address all the fistula consequences at the same time. Additionally, strengthening the health care system through; establishment and facilitation of new fistula centers, staff capacity building, reproductive health education to address knowledge and attitudes, and strengthening patient follow-up will greatly facilitate the reintegration process and restoration of women dignity.

## Additional file

Additional file 1: Interview guide - Post effects of Fistula among Fistula Survivors. (PDF 115 kb)

## Data Availability

The datasets used and/or analysed during the current study are available from the corresponding author on reasonable request.

## Abbreviations

MoH: Ministry of Health
OF: Obstetric fistula
UBOS: Uganda Bureau of Statistics
UDHS: Uganda Demographic Health Survey
UNFPA: United Nations Population Fund
WHO: World Health Organization
