# Supplementary material for: Post - effects of obstetric fistula in Uganda; a case study of fistula survivors in KITOVU mission hospital (MASAKA), Uganda
Source: BMC Public Health. 2019 Jun 6;19:696. doi: 10.1186/s12889-019-7023-7 (PMC6555098; doi:10.1186/s12889-019-7023-7)
Supplement: Supplementary file 1 — Interview guide - Post effects of Fistula among Fistula Survivors. (PDF 115 kb) [file 12889_2019_7023_MOESM1_ESM.pdf]

POST - EFFECTS OF OBSTETRIC FISTULA IN UGANDA; A CASE STUDY OF FISTULA SURVIVORS IN KITOVU MISSION HOSPITAL (MASAKA), UGANDA

INTERVIEW GUIDE FOR FISTULA SURVIVORS

(An interview guide to be used on women with treated fistula)

District…………………………………………………………………………..

LC II Zone……………………………….…………………………………….

Name of Health Facility ………………………………………………………

Introduction:

Miss/Mrs. ………………………………………………………………………………

I am called Nakilembe Jennifer, a student at Makerere University pursuing a Master's degree of science in Reproductive Health. I am collecting information on Post-effects of obstetric fistula among women with treated fistula. I very much appreciate your participation in this research study. Your participation is purely voluntary and the information you provide will be kept strictly confidential and will not be shown to any other person. Participating in this study has no risks involved and may not give you personal benefit but may go a long way into understand the experiences that fistula survivors go through. You are free to opt out of this study any point in time when you feel uncomfortable or otherwise.

Do you agree or consent to participate in this study? Yes………, No ………….

If so, please sign or thumb print …………….

Interview started at …………………………………..am/pm

Interview ended at …………………………………...am/pm

I would like you to introduce yourself to me, by telling me your names, age, marital status, level of education, residence and your religion.

1. What do you do for a living?
2. How did you know that you had fistula?
3. When did you get the fistula and how old were you?
4. In your opinion what might have caused it?
5. For how long did you live with it (years/months)?
6. Who knew that you had fistula?
7. What was the reaction of those who knew about your condition? (Probe on how she felt)
8. How did you find out that it was treatable?
9. When was the fistula operated on? (Probe for the time she spent with it and why so)
10. How successful was the operation?
11. Since the operation, how is your personal health (probe for also the social life)
12. What difference do you have before and after the fistula repair?
13. What was the family' reaction when you returned after the successful operation?
14. What has been done to ensure that you do not continue the negative experiences you had before?
15. What has the “successful “operation meant to you as a person?

16. And what difference has it made in your life?

17. What more should be done;

i. To prevent OF
ii. Enable affected people access help
iii. Facilitate re-integration

END
